# Effects of concurrent and staggered dosing of semi-solid enteral nutrients on pharmacokinetic behavior of antiepileptic drugs after oral administration in rats

**DOI:** 10.1371/journal.pone.0259400

**Published:** 2021-11-09

**Authors:** Katsuhito Nagai, Yoshikazu Ryuno, Yoshihito Iwanami, Sachiko Omotani, Shuhei Fukuno, Yasutoshi Hatsuda, Hiroki Konishi, Michiaki Myotoku

**Affiliations:** 1 Faculty of Pharmacy, Osaka Ohtani University, Tondabayashi, Osaka, Japan; 2 Research and Development Division, EN Otsuka Pharmaceutical Co., Ltd., Tiyoda-ku, Tokyo, Japan; Belgrade University Faculty of Medicine, SERBIA

## Abstract

**Background:**

The use of enteral nutrients plays a highly important role in accurate nutrition management, but limited information is currently available on the cautionary points of semi-solid enteral nutrients.

**Aim:**

In this study, we examined whether the pharmacokinetic profiles of sodium valproate (SVA), levetiracetam (LEV), and carbamazepine (CBZ) are affected by altering the dosing time of RACOL^®^-NF Semi Solid for Enteral Use (RASS), a prescribed semi-solid formula. We also investigated whether the pharmacokinetic interaction observed in this study can be avoided by staggered dosing of the chemical drug and semi-solid enteral nutrient.

**Methods:**

The plasma concentration of SVA, LEV and CBZ after oral administration was measured by LC-MS/MS method.

**Results:**

There was no difference in pharmacokinetic characteristics of SVA and LEV when the dosing time of RASS was altered. On the other hand, the plasma concentration of CBZ after oral administration at all sampling points decreased with the extension of the dosing time of RASS, which was consistent with the C_max_ and AUC. However, no significant difference was observed in the pharmacokinetic profiles or parameters of CBZ between the short-term and long-term RASS dosing groups by prolonging the administered interval of CBZ and RASS for 2 hr.

**Conclusion:**

We concluded that the pharmacokinetic profiles of CBZ, but not SVA and LEV, after its oral administration are affected by the dosing time of RASS, but staggered administration of CBZ and RASS prevented their interaction.

## Introduction

Sufficient feeding is essential for critically ill patients, and helps to prevent malnutrition and accompanying complications [1, 2]. Feeding *via* a percutaneous endoscopic gastrostomy (PEG) tube is a safe and efficient approach for patients who cannot maintain a sufficient oral intake. Liquid and semi-solid products are commercially available as enteral nutrients, and have are frequently used for nutritional management. However, the use of liquid enteral nutrients is occasionally accompanied by adverse effects, which develop over a long clinical course. Diarrhea is the most common adverse effect reported in patients receiving liquid enteral nutrients, and occurs in up to 30% of patients in medical and surgical wards [3]. Feeding is also complicated by gastroesophageal reflux disease, resulting in aspiration-induced pneumonia, the prevalence of which is in the range of 10–22% [4, 5]. The gastric complications caused by liquid enteral nutrients are a serious problem worldwide, as demonstrated in the guidelines of the organizations associated with nutrition management in each country. The semi-solidification of enteral nutrients is available as a strategy to prevent the adverse events associated with liquid enteral nutrients [6, 7]. However, limited information is currently available on the cautionary points of semi-solid enteral nutrients such as drug-drug interactions.

Therapeutic agents can generally be used safely and effectively by maintaining the blood concentration of the agent within the effective therapeutic index. Several antiepileptic drugs and antiarrhythmic drugs possess a particularly narrow effective treatment index. Blood concentrations of these drugs are monitored regularly and the dose is determined based on the results. Pharmacokinetic interactions have been recently reported between enteral nutrients and antiepileptic drugs, such as phenytoin, which have a narrow therapeutic index [8]. The previous studies demonstrated that the pharmacokinetic behaviors of several drugs with a narrow therapeutic index are affected by the diet [9, 10]; however, the dosing interval between the semi-solid enteral nutrients and chemical drugs varied among medical institutions and staff, which resulted in the high possibility of pharmacokinetic interaction between these drugs [11]. In addition, the dosing time of semi-solid nutrients is generally different based on the disease conditions. The gastric emptying rate (GER) and gastric pH were reported to be influenced by feeding time and dietary lipids [12, 13], which is resulted in altered absorption efficacy of chemical drugs [14, 15]. Therefore, it is possible that the blood concentration of chemical drugs was influenced by the dosing time of semi-solid enteral nutrients as with meals.

Our previous study demonstrated that the pharmacokinetics of the antiepileptic drug carbamazepine (CBZ) are altered by concomitant use with sodium alginate (SA) [16]. As RACOL^®^-NF Semi Solid for Enteral Use (RASS) is a product that contains SA and agar in addition to nutritional components, and has a viscosity of 6,500–12,500 mPa·sec, pharmacokinetic interactions may occur between RASS and several antiepileptic drugs. The aim of this study was to clarify whether the pharmacokinetic profiles of three narrow-therapeutic index antiepileptic drugs, including CBZ, are affected by altering the dosing time of RASS. We also investigated whether the pharmacokinetic interaction observed in this study can be avoided by staggered dosing of the drug and semi-solid enteral nutrients.

## Materials and methods

### Chemicals

Sodium valproate (SVA) was purchased from Kyowa Hakko Kirin Co., Ltd (Tokyo, Japan). Levetiracetam (LEV) was purchased from Otsuka Pharmaceutical Co., Ltd (Tokyo, Japan). CBZ was purchased from Mitsubishi Tanabe Pharm Corporation (Osaka, Japan). RASS was obtained from EN Otsuka Pharmaceutical Co. Ltd (Iwate, Japan). All other reagents were of commercial or analytical grade, requiring no further purification.

### Animal care and treatment

Male Sprague-Dawley rats, aged 8 weeks, were purchased from Charles river laboratories Japan, Inc. (Kanagawa, Japan). Rats were acclimatized for 1 week before placement of the gastric catheter. Rats were fasted for 16 hr with free access to water and then an intragastric catheter was placed under the anesthetized condition by isoflurane. To alleviate suffering, intramuscular injection of buprenorphine was conducted after the operation as pain control. The joint between the gastric catheter and the nutrient infusion pump was made free-moving, which reduced restraint stress during administration of the nutrient. The recovery period was 3 days from the day of intragastric catheter. After recovery, SVA (100 mg / kg), LEV (100 mg/kg), and CBZ (50 mg / kg) were orally administered to rats just before RASS *via* an intragastric catheter. RASS was administered at a dose of 10 g over 30 min (short-term group) or 2 hr (long-term group) based on previous reports [17–19]. In the case of SVA, serial blood samples were obtained from the tail vein 15, 30, and 45 min, and 1, 1.5, 2, and 3 hr after oral administration of the drug. In the case of LEV and CBZ, serial blood samples were obtained from the tail vein 15, 30, and 45 min, and 1, 1.5, 2, 3, 4, and 8 hr after oral administration of these drugs. After the last blood sampling, all rats were euthanized by inhalation of carbon dioxide gas. Blood was centrifuged (2,000 *g*, 5 min, 4°C) to obtain plasma. For CBZ, a pharmacokinetic study was also conducted in which RASS was started 2 hr after the administration of CBZ. The animal experiment in the present study was carried out in Kamakura Techno Science Co., Ltd., contracted from EN Otsuka Co., Ltd. The experimental protocols and animal care methods used in the present study were approved by the Committee on Animal Research and Ethics at Kamakura Techno Science Co., Ltd.

### Measurement of plasma concentrations of three drugs

The plasma concentration of each drug was measured by LC-MS/MS based on the ESI (electrospray ionization) method. The LC-MS/MS system consisted of a high-performance liquid chromatograph LC-30AD system (Shimadzu, Kyoto, Japan) and an AB Sciex QTRAP 6500 tandem mass spectrometer (Framingham, MA, USA). Capcell Pak C18 MGII (particle size 5 μm, inner diameter 2.0 mm x 50 mm; Osaka Soda, Osaka, Japan) was used as the separation column. To measure the concentration of SVA, solvent A (water containing 0.063% ammonium formate) and solvent B (acetonitrile) were used as the mobile phase. The gradient conditions were maintained at 20% solvent B for 1.5 min, increased from 20% to 95% solvent B in 1.50 min, held at 95% for 1.0 min, decreased back to 20% within 0.01 min, and held at 20% for 1.49 min. To measure the concentration of LEV and CBZ, solvent A (water containing 0.1% formic acid) and solvent B (acetonitrile) were used as the mobile phase. The gradient conditions of LEV were increased from 5% to 60% solvent B in 1.50 min, sharply increased to 95% within 0.01 min, held at 95% for 1.49 min, decreased back to 5% within 0.01 min, and held at 5% for 1.49 min. The gradient conditions of CBZ were increased from 30% to 95% solvent B in 2.00 min, held at 95% for 1.00 min, decreased back to 30% within 0.01 min, and held at 30% for 1.49 min.

### Pharmacokinetic analysis

Pharmacokinetic analysis was performed by a non-compartmental model using Phoenix WinNonlin *ver* 7.0 (Certara, Princeton, NJ, USA). The elimination rate at the terminal phase (k_el_) was determined by linear regression of the log-linear portions of plots of plasma concentration against time. AUC_0→∞_ was obtained by extrapolating to infinity using the ratio of the last measured concentration to k_el_.

### Statistical analysis

Data are expressed as means ± S.D. Differences between the means of two groups were compared using the Student’s unpaired *t*-test or Aspin-Welch *t*-test. Differences with a *p*-value of 0.05 or less were considered significant.

### Ethics

This study was jointly conducted by Osaka Ohtani University and EN Otsuka Pharmaceutical Co., Ltd. The animal experiment in the present study was carried out in Kamakura Techno Science Co., Ltd., contracted from EN Otsuka Pharmaceutical Co., Ltd. The experimental protocols and animal care methods used in the present study were approved by the Committee on Animal Research and Ethics at Kamakura Techno Science Co., Ltd. The purpose of this study is to obtain information for proper nutritional management for patients with drug treatment and does not claim the superiority or safety of RASS.

## Results

### Pharmacokinetics of SVA, LEV, and CBZ in combination with RASS

**Fig 1:**
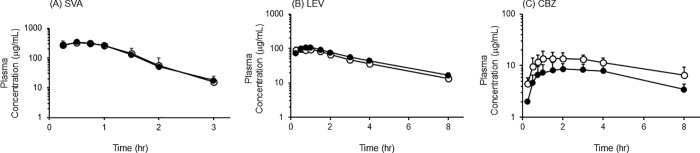
Serum concentration-time courses of SVA, LEV, and CBZ after concurrent oral administration with RASS. Serum concentrations of SVA (A), LEV (B), and CBZ (C) were measured after oral administration. Results are shown as the means ± SD of five rats per group. Open circles: short-term group; Closed circles: long-term group.

**Fig 2:**
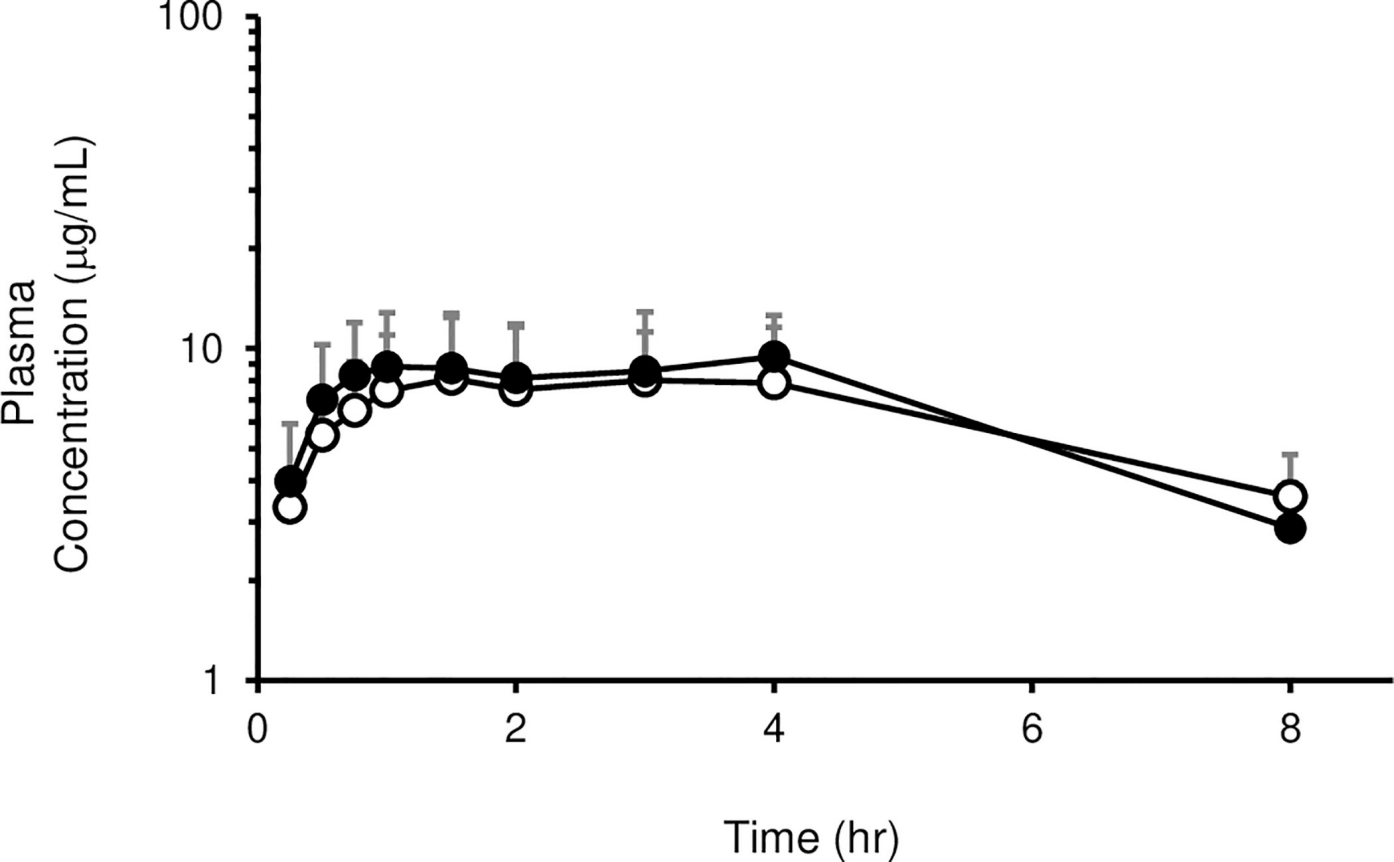
Serum concentration-time courses of CBZ after staggered oral administration with RASS. Serum concentrations of CBZ were measured after oral administration with RASS. Results are shown as the means ± SD of five rats per group. Open circles: short-term group; Closed circles: long-term group.

The plasma concentration-time curves of each drug are shown in Figs 1 and 2, and Tables 1–4. There was no difference in pharmacokinetic characteristics of SVA or LEV between the short-term and long-term groups (Fig 1A and 1B, Tables 1 and 2). On the other hand, the plasma concentration of CBZ at all blood sampling points in the long-term group was lower than that in the short-term group (Fig 1C). In addition, the values of C_max_ and AUC_0-480 min_ were significantly lower in the long-term group than in the short-term group (Table 3). However, no significant difference was observed in the pharmacokinetic profiles, C_max_, or AUC_0→480 min_ of CBZ between the short-term and long-term groups by prolonging the dosing interval of CBZ and RASS for 2 hr (Fig 2, Table 4).

**Table 1:** Pharmacokinetic parameters of concurrent administration of SVA and RASS.

	Short-term group	Long-term group
C_max_ (μg/mL)	340 ± 75	352 ± 66
T_max_ (h)	0.65 ± 0.22	0.50 ± 0.030
AUC_0→180 min_ (μg•hr/mL)	452 ± 89	446 ± 80
AUC_0→∞_ (μg•hr/mL)	466 ± 83	464 ± 76
MRT (hr)	1.0 ± 0.2	1.1 ± 0.2
K_el_ (1/h)	1.4 ± 0.4	1.4 ± 0.4
t_1/2_ (h)	0.55 ± 0.15	0.56 ± 0.22
K_a_ (1/h)	2.0 ± 0.5	2.1 ± 0.0

Results are shown as the means ± SD of five rats per group.

**Table 2:** Pharmacokinetic parameters of concurrent administration of LEV and RASS.

	Short-term group	Long-term group
C_max_ (μg/mL)	102 ± 7	110 ± 8
T_max_ (h)	0.60 ± 0.34	0.80 ± 0.21
AUC_0→480 min_ (μg•hr/mL)	361 ± 45	414 ± 36
AUC_0→∞_ (μg•hr/mL)	414 ± 53	483 ± 54
MRT (hr)	3.8 ± 0.4	4.1 ± 0.5
K_el_ (1/h)	0.26 ± 0.03	0.25 ± 0.04
t_1/2_ (h)	2.7 ± 0.3	2.9 ± 0.4
K_a_ (1/h)	5.8 ± 2.5	2.9 ± 0.6

Results are shown as the means ± SD of five rats per group.

**Table 3:** Pharmacokinetic parameters of concurrent administration of CBZ and RASS.

	Short-term group	Long-term group
C_max_ (μg/mL)	15.1 ± 4.1	9.07 ± 2.73*
T_max_ (h)	2.0 ± 1.2	2.5 ± 1.0
AUC_0→480 min_ (μg•hr/mL)	84.0 ± 15.3	51.7 ± 12.0**
AUC_0→∞_ (μg•hr/mL)	146 ± 79	132 ± 104
MRT (hr)	8.7 ± 7.6	18 ± 22
K_el_ (1/h)	0.19 ± 0.09	0.17 ± 0.15
t_1/2_ (h)	5.6 ± 5.3	12 ± 16
K_a_ (1/h)	1.5 ± 0.6	0.92 ± 0.63

Results are shown as the means ± SD of five rats per group.

* *p*<0.05 *vs* the short-term group

** *p*<0.01 *vs* the short-term group.

**Table 4:** Pharmacokinetic parameters of staggered administration of CBZ and RASS.

	Short-term group	Long-term group
C_max_ (μg/mL)	8.46 ± 4.52	10.8 ± 2.73
AUC_0→480 min_ (μg•hr/mL)	51.2 ± 26.5	56.6 ± 13.8

Results are shown as the means ± SD of five rats per group.

## Discussion

Enteral nutrients play a highly important role in improving nutritional management. Many fundamental and clinical studies have been conducted on the satisfactory use of enteral nutrients. This study focused on pharmacokinetic interactions between antiepileptic drugs and semi-solid enteral nutrients.

The pharmacokinetics of SVA and LEV were not affected by the dosing time of RASS. As shown in Table 3, individual differences were observed in the pharmacokinetics of CBZ among rats. The observation was probably due to the difference in solubility of CBZ in gastrointestinal fluid, and/or the expression level and activity of cytochrome P450 CYP3A, a metabolizing enzyme of the drug [20, 21]. However, when RASS was simultaneously dosed for a short period, the plasma concentration of CBZ was significantly higher than that with long-term dosing, which was consistent with the C_max_ and AUC. Among the drugs selected in this study, only CBZ is fat-soluble and bile was reported to increase its solubility in the intestinal tract [22]. Although there is no gallbladder in rats, the previous study demonstrated that the ability to synthesize bile acids was increased by feeding [23]. Bile secretion may be activated by short-term dosing of RASS because of the transient increase in the amount of food ingredients that pass through the stomach and gastrointestinal tract. The opportunity of contact between the fat component in RASS and CBZ may be increased by short dosing. As a result, the solubility of CBZ in the gastrointestinal tract was increased, which is responsible for the altered absorption efficiency of the drug. In addition, fiber substances contained in enteral nutrients were reported as a factor that alters the serum concentration of CBZ [24]. Therefore, it may be necessary to pay attention to alteration in the blood concentrations of chemical drugs when switching between liquid and semi-solid enteral nutrients or altering administration time of the nutrients in patients receiving fat-soluble drugs with a narrow therapeutic index.

The T_max_ of CBZ is 0.50–2.58 hr in normal rats [20, 25], suggesting that the orally administered dose of CBZ was almost completely absorbed in the absence of RASS within this time frame. As shown in Fig 2 and Table 4, no effects of dosing time of RASS on the CBZ pharmacokinetic profiles were observed by prolonging the dosing interval of these drugs for 2 hr. Therefore, staggered dosing should be generally applicable to the combined use of oral drugs with semi-solid enteral nutrients in clinical practice by understanding the absorption rate of the drugs. However, in the case of some drugs, such as hypoglycemic agents, serious adverse events, such as hypoglycemia, may be caused by extending the interval between meals and drug administration. Thus, it is important to give guidelines on appropriate administration timing between chemical drugs and enteral nutrients based on the characteristics of each drug.

Many drugs have been reported to interact with food [9, 10], but few have been reported to interact with enteral nutrients. In addition to the secreted bile and dietary lipids mentioned above, protein and calcium which are generally compounded into the enteral nutrients could be a factor for occurring pharmacokinetic interaction between chemical drugs and diets [26, 27]. Since the enteral nutrients are similar to diets in their nutritional compositions, they would influence on GER and gastric pH as with diets, followed by alteration in absorption efficacy of chemical drugs. Nevertheless, there is no clinical information on the pharmacokinetic interaction between chemical drugs and semi-solid eternal nutrients. The dose of RASS used in this study was determined based on the recommendations of the maximum permissible dosage from the European Federation of Pharmaceutical Industries and Associations (EFPIA) and the EURL ECVAM Database on Alternative Methods (ECVAM). In this regard, pharmacokinetic interactions between CBZ and semi-solid enteral nutrients are considered to occur easily in clinical practice, thus further clinical examinations are needed.

## Conclusion

In conclusion, we demonstrated using rats that the pharmacokinetic profiles of CBZ, but not SVA and LEV, after its oral administration are affected by the dosing time of RASS, but staggered administration of CBZ and RASS prevented their interaction. Our study will help to promote appropriate nutritional management in pharmacotherapy.
